# Integrated network pharmacology, molecular docking, and animal experiments to reveal the potential mechanism of hesperetin on COPD

**DOI:** 10.1038/s41598-025-95810-4

**Published:** 2025-04-01

**Authors:** Jingxi Wang, Hongyang Wang, Xin Kang, Xiaotian Wang, Xi Li, Jie Guo, Xuan Jing, Xi Chu, Xue Han

**Affiliations:** 1 The First Affiliated Hospital, Hebei University of Chinese Medicine, Shijiazhuang, China; 2 Hebei Industrial Technology Institute for Traditional Chinese Medicine Preparation, Shijiazhuang, China; 3 Hebei University of Chinese Medicine, Shijiazhuang, China; 4 The Fourth Hospital of Hebei Medical University, Shijiazhuang, China; 5 School of Pharmacy, Hebei University of Chinese Medicine, Shijiazhuang, China

**Keywords:** Chronic obstructive pulmonary disease, Hesperetin, Network pharmacology, Molecular docking, MAPKs/NF-κB signaling pathway, Drug discovery, Diseases, Medical research

## Abstract

Hesperetin (HE), a natural flavonoid exhibiting anti-inflammatory and antioxidant properties, holds significant potential in treating chronic obstructive pulmonary disease (COPD). Nonetheless, the precise mechanisms underlying its effects are yet to be fully elucidated. In this study, we aim to explore the role and potential mechanism of HE in treating COPD using network pharmacology, molecular docking and experimental validation. We screened for HE and COPD-related targets from public databases, and then imported potential targets into a STRING database to establish a protein–protein interaction network. Gene ontology (GO) and Kyoto encyclopedia of genes and genomes enrichment analysis were performed to obtain key signaling pathways. We then predicted the binding interactions between HE and core targets using molecular docking. The animal model of COPD was established through lipopolysaccharide and cigarette smoke induction in mice to observe lung function, inflammatory factors, pathology, and the expression of related proteins. Network pharmacology findings unveiled that HE and COPD shared 105 common targets. MAPKs and NF-κB signaling pathways were selected for further validation. In animal experiment, HE enhanced lung function and histopathological morphology, while reducing inflammation levels. The results of Western blot tests indicated that HE treatment considerably inhibited the expression of MAPKs and NF-κB. HE effectively reduced lung inflammation and improved lung function in mice. This mechanism may be achieved by inhibition of MAPKs and NF-κB signaling pathways.

## Introduction

Chronic obstructive pulmonary disease (COPD) is a diverse pulmonary disease characterized by chronic airway inflammation and structural obstruction of the respiratory tract^1^. According to the latest report from the WHO, COPD ranks among the top three leading causes of death worldwide^2^. Many patients suffer from COPD and die prematurely as a result of the disease or its complications. Globally, as ongoing exposure to COPD risk factors persists and the population ages, the impact of COPD is projected to significantly increase in the coming decades^3,4^. A substantial body of meta-analyses and systematic reviews have provided evidence of strong associations between factors such as smoking, age, and oxidative stress and COPD^5–7^. Currently, many therapies can improve pulmonary function, reduce exacerbations, and enhance life quality in COPD patients. However, these come at the cost of considerable economic and social burden^8–10^.

Roflumilast (ROF), a Phosphodiesterase-4 (PDE4) inhibitor, has been shown to decrease the number of moderate to severe acute exacerbations in patients suffering from chronic bronchitis and advanced stages of COPD, particularly in individuals with a history of exacerbations that necessitated systemic corticosteroid therapy^4^. However, the detrimental effects of ROF on COPD are more frequent and costly^11,12^. The most common are nausea, diarrhea, abdominal pain, diminished appetite, weight loss, headache, and sleep disturbance, all of which limit its clinical usage^4^.

Due to their lower side effects and increased safety, natural medicines are receiving more and more attention. As a natural flavonoid, hesperetin (HE) can combat respiratory illnesses by bolstering the spleen, managing qi, dissolving phlegm, drying dampness, and assuaging cough^13,14^. In contrast to bronchodilators and glucocorticoids, no drug resistance or adverse reactions have been reported in HE’s treatment of COPD^14^. Recent advances in pharmacological research have shown that HE exhibits anti-inflammatory, antioxidant, and antitumor properties^15^, underlining its potential as a viable therapeutic strategy for COPD. Simultaneously, HE exhibited a notable antioxidant effect in comparison to naringenin^16^. However, the precise mechanisms by which HE produces its effects in COPD treatment remain unclear. If its potential mechanism is validated, it could bring new hope for the treatment of COPD.

The pathogenesis of COPD involves a complex interplay of inflammatory signaling pathways, among which the mitogen-activated protein kinases (MAPKs) and nuclear factor-kappa B (NF-κB) pathways are central players^17^. The crosstalk between MAPKs and NF-κB pathways is crucial in modulating the inflammatory response in COPD. For example, TAK1 serves as an upstream kinase for both NF-κB and JNK signaling, suggesting a convergence point for these pathways^18^. Additionally, NF-κB can inhibit JNK-mediated apoptosis, highlighting the complex interplay between these pathways in regulating cell survival and inflammation. This crosstalk is further supported by studies showing that inhibition of either pathway can attenuate inflammation and oxidative stress in COPD models^19^.

Modern studies primarily investigate the mechanism of drug treatment by establishing COPD animal models^20^. Currently, methods with high success rates include smoking combined with lipopolysaccharide (LPS), smoking alone^21^, smoking combined with protease instillation, and others^22^. LPS, more commonly known as endotoxin, is found in the cell walls of Gram-negative bacteria^23^, and acts as a microbial component that elicits an inflammatory response^24^. LPS might modify the inflammatory immune response within the lungs, potentially contributing to, or exacerbating, the progression of COPD^25,26^. We created a COPD mouse model by exposing mice to cigarette smoke and LPS to study the impact of drugs on lung inflammation.

**Fig. 1:**
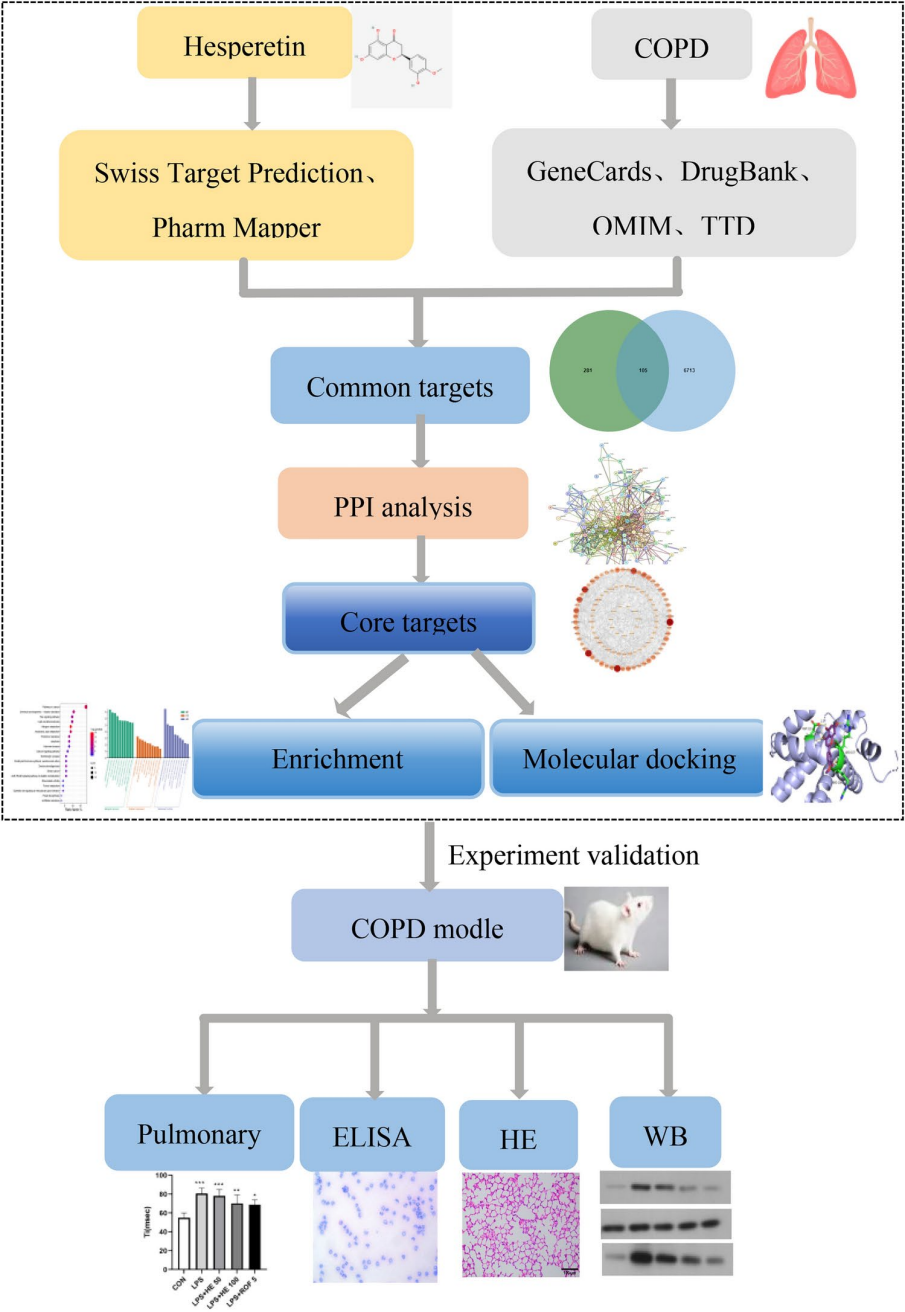
The detailed research process.

As an emerging method in drug research, network pharmacology can uncover potential targets between drugs and diseases by analyzing the action network of these drugs. Protein interaction networks can predict and evaluate mechanisms^27^. Therefore, with the research process shown in Fig. 1, we utilize network pharmacology, molecular docking, and animal studies to examine and validate possible targets and mechanisms of HE in treating COPD.

## Methods and materials

### Network pharmacology analysis

#### Acquiring targets of HE and COPD

**Fig. 2:**
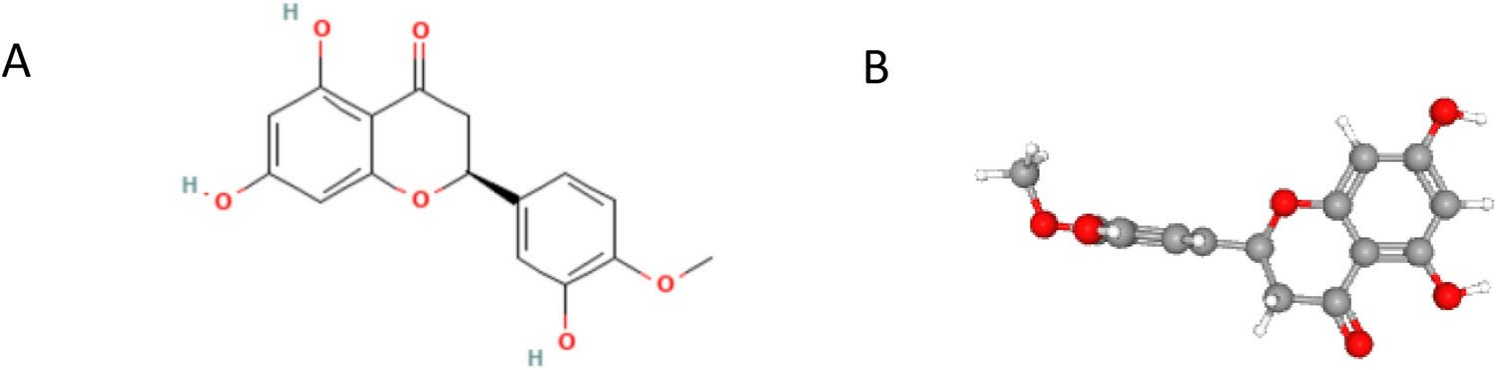
(**A**) 2D structure of HE. (**B**) 3D structure of HE.

To obtain the 2D structure (Fig. 2A), 3D structure (Fig. 2B), and SMILES information, download them from the PubChem database (https://pubchem.ncbi.nlm.nih.gov/)^28^. Next, respective SMILES numbers must be input into the Swiss Target Prediction platform (http://swisstargetprediction.ch)^29^ and the probability > 0 was selected to screen potential effective targets. HE was retrieved from the Pharm Mapper platform (https://lilab-ecust.cn/pharmmapper/)^30^ for the prediction of HE component targets (Supplementary Table 1). COPD gene targets were predicted using four databases: GeneCards (https://www.genecards.org/)^31^, DrugBank^28,32^, OMIM(https://omim.org/)^33^, and TTD (https://db.idrblab.net/ttd/)^34^ (Supplementary Table 2). The search term “chronic obstructive pulmonary disease” was utilized to identify and predict disease targets associated with COPD.

#### Protein–protein interaction

Online Venn analysis software was utilized to identify overlapping target genes associated with both HE and COPD^35^. The protein interaction data of HE for COPD were analyzed in the STRING database (https://cn.string-db.org/)^36^. The downloaded TSV files were then imported into Cytoscape 3.9.0 for PPI network visualization and analysis. Hub genes were identified from the gene network using the Betweenness centrality metric, with the median value of this parameter serving as the selection criterion in each screening step.

#### GO and KEGG analysis

We submitted the intersecting targets to the Metascape database (https://metascape.org/)^37^ for GO and KEGG (https://www.kegg.jp/kegg/kegg1.html) functional enrichment analysis^38^. Pathway terms with P-values less than 0.05 were considered significant.

#### Molecular docking

Molecular docking was conducted between HE and the six top-ranked target proteins identified in the PPI network. The 2D structure SDF files of HE, sourced from the PubChem database, were imported into Open Babel software and converted to PDB format. The 3D crystal structure of the target protein, in PDB format, was retrieved from the PDB database (https://www.rcsb.org/)^39^. PyMOL 2.4.0 was used to remove water molecules and ligands from the protein structure^40^. For docking studies, protein structures were prepared by adding nonpolar hydrogens and converting the structures to PDBQT format. Parameters for spacing were set to locate the active pocket for protein–ligand docking. Finally, Autodock Vina 1.1.2 software was used for docking, where lower binding energy signifies greater stability. The results presenting lower binding energy were visualized using the PyMOL software.

### Chemical reagents

The chemical reagents HE and ROF were obtained from Shanghai Macklin Biochemical Technology Co., Ltd. LPS was acquired from Sigma-Aldrich Trading Co., Ltd (Shanghai, China). The cigarettes used in the smoking experiment were Honghe brand cigarettes manufactured by the Hongyun Honghe Group. All the chemical reagents used for the Western Blot (WB) procedure (Whole protein extraction kit, WLA019; BCA Protein Concentration Assay Kit, WLA004; Western wash solution, WLA025; WB blocking solution, WLA066; ECL luminescent solution, WLA006) were purchased from Shenyang Wanlei Biotechnology Co., Ltd. Unless stated otherwise, all the other analytical-grade reagents used in this study were provided by Shanghai McLean Biochemical Technology Co., Ltd.

### Animal experiments

#### Experimental animals

Forty SPF-grade, healthy male (C57BL/6J) mice (6-week-old, weighing 20 ± 2 g) were selected. These were provided by Jiangsu Huachuang Cigna Medical Technology Co., Ltd (License number: SCXK(SU)2020-0009). These mice were housed in an environment following a 12-h light/dark cycle, with an ambient temperature maintained at 22 ± 1 °C and humidity levels between 45 and 55%. Within a standard laboratory environment, the mice had unlimited access to food and water. Following a week of acclimatization, the experimental model was established. This study obtained approval from the Animal Health and Ethics Committee of the Hebei Provincial Hospital of Traditional Chinese Medicine (Shijiazhuang, China; Approval No. DWLL202306078). All experimental protocols adhered to the Guidelines for the Care and Use of Laboratory Animals.

#### Experimental design

Forty mice were divided into five groups by random number table method (n = 8): the blank control group (CON group), the model group (LPS group), a low-dosage hesperetin group (LPS + HE 50 mg/kg), a high-dosage hesperetin group (LPS + HE 100 mg/kg), and the positive control group (LPS + ROF 5 mg/kg). The COPD mouse model was created via intranasal inhalation of LPS (7.5 μg/50 μL saline) on days 0 and 14. Excluding the days when LPS was administered, the mice were subjected to cigarette smoke exposure (nine cigarettes per hour, for 2 h each session, twice daily, 6 days a week), from day 0 to day 60. Every cigarette contained 11 mg of tar, 1 mg of nicotine, and 13 mg of CO. From day 20 to day 60, mice in the CON group were exposed to room air and given an equal volume of saline by gavage. LPS + HE 50 group: the mice were daily administered HE by gavage at a dose of 50 mg/kg. LPS + HE 100 group: the mice were daily administered HE by gavage at a dose of 100 mg/kg. LPS + ROF 5 group: Roflumilast (5 mg/kg) was administered by gavage daily^14,41–43^.

#### Evaluation of pulmonary function

The Inspiratory Time (TI), Maximal Inspiratory Volume (PIF), Tidal Volume (TV), End-Expiratory Pause (EEP), Expiratory Time (ET), Maximal Expiratory Volume (PEF), Expiratory Volume (EV), End-Inspiratory Pause (EIP), Relaxation Time (RT), Inspiratory Volume per Minute (MV), Respiratory Rate (F), 50% Expiratory Flow Rate (EF50), and Forced Breathing Interval (PENH) were measured with a pulmonary function instrument.

#### The number of inflammatory cells

After successful modeling, the mice were euthanized with CO_2_ on day 60, and bronchoalveolar lavage fluid (BALF) samples were collected. Cell precipitation was obtained by centrifugation at 300 × g for 10 min. The cells were then fixed on slides for Giemsa staining (item number D010, Nanjing JianCheng Bioengineering Institute, China). Afterward, the quantities of inflammatory cells, neutrophils, and total mononuclear cells were determined with the aid of a light microscope.

#### Levels of inflammatory factors in BALF

The pro-inflammatory cytokines in the BALF were assessed using an enzyme-linked immunosorbent assay (ELISA). The levels of IL-6, IL-1β, and TNF-α were determined utilizing an ELISA Kit (Shenyang WanLei Biotechnology Co., Ltd., Shenyang, Liaoning Province, China).

#### Measurement of NE activity and ROS levels

A 0.1 ml sample of BALF was incubated at 37 °C for 2 h with 0.5 M succinyl-(Ala)3-p-nitroanilide. The reaction was then terminated by adding 2 ml of 10% trichloroacetic acid. The precipitate was removed through centrifugation, performed at 3000 revolutions per minute for 10 min. A 3 ml portion of the supernatant was combined with 0.4 ml of 0.1% NaNO_2_ to initiate the diazotization reaction. Any excess NaNO_2_ was neutralized by adding 0.5 ml of 0.5% ammonium sulfamate. The reaction was colored by adding 0.4 ml of 0.1% N-1-naphthalene diamine dihydrochloride (10% ethanol). Neutrophil elastase (NE) activity in the BALF was assessed by measuring the absorbance of the diazo compound at 405 nm.

DCFH-DA (10 μM) was added to the single-cell suspension and incubated at 37 °C for 30 min. Subsequently, the single-cell suspension was harvested and centrifuged at 1000 g for 10 min. The supernatant was discarded, and the cells were resuspended in phosphate buffered saline (PBS). Finally, the intracellular reactive oxygen species (ROS) content was measured using a fluorescence microplate reader.

#### Histopathological analysis

The harvested lung tissues were preserved in a 4% paraformaldehyde solution for 48 h. The fixed tissue samples underwent sequential processes, including dehydration, wax infiltration, embedding, sectioning, baking, and deparaffinization. Subsequently, they were stained using Hematoxylin and eosin (H&E) staining solution, and the slides were mounted with neutral gum. Ultimately, the stained sections were examined microscopically.

#### Immunohistochemical analysis

Lung tissue sections were dehydrated and embedded in paraffin wax, after which antigen retrieval was performed using a citric acid buffer. These sections were then incubated with 3% H₂O₂ for 15 min at room temperature. The primary antibody, diluted to a 1:100 concentration with PBS, was left to incubate overnight at 4 °C. The tissue sections’ incubation with a horseradish peroxidase-conjugated secondary antibody took place at 37 °C for 1 h. The color was initially developed with Diaminobenzidine (DAB), counterstained with hematoxylin for 3 min, dehydrated with absolute ethanol, made transparent with xylene, mounted with neutral gum, and then examined under a microscope.

#### Western blot analysis

The pyrolysis solution was pre-melted at room temperature, and the projected volume for the experiment was divided into PMSF, with 1% of the sectioned volume. Subsequently, based on the mass and volume of each sample, the corresponding volume of the lysed sample was added. After a 10-min pause, the supernatant was separated into the resultant protein extract. The entire protein content was then quantified using a commercial BCA assay kit. The total protein was then separated via SDS-PAGE gel electrophoresis and subsequently blotted onto a polyvinylidene fluoride (PVDF) membrane. The membrane was rinsed once with TBS with Tween-20 (TBST), followed by gentle agitation in a blocking solution on a shaker for 1 h. The membranes were then incubated overnight at 4 °C with primary antibodies against p-NF-κB p65, NF-κB p65, p-ERK1/2, ERK1/2, p-JNK, JNK, p-p38, p38, and β-actin. After four TBST washes, the membranes were incubated with the corresponding secondary antibodies at room temperature for 30 min. Lastly, the optical density of the target bands was analyzed using a gel imaging system equipped with Gel-Pro-Analyzer software following exposure to the ECL luminescent solution in a darkroom.

#### Statistical analysis

For the analysis of data across multiple groups, One-Way analysis of variance (ANOVA) was conducted, followed by Tukey’s post-hoc test to perform pairwise comparisons using Graphpad Prism software. The findings for the three groups are reported as mean ± standard deviation (SD). A *p* value below 0.05 was considered indicative of statistical significance.

### Ethics statement

We confirmed that all animal experiments complied with the ARRIVE guidelines and were reviewed and approved by the Animal Health and Ethics Committee of Hebei Provincial Hospital of Traditional Chinese Medicine (DWLL202306078).

## Results

### Potential targets and PPI network analysis

**Fig. 3:**
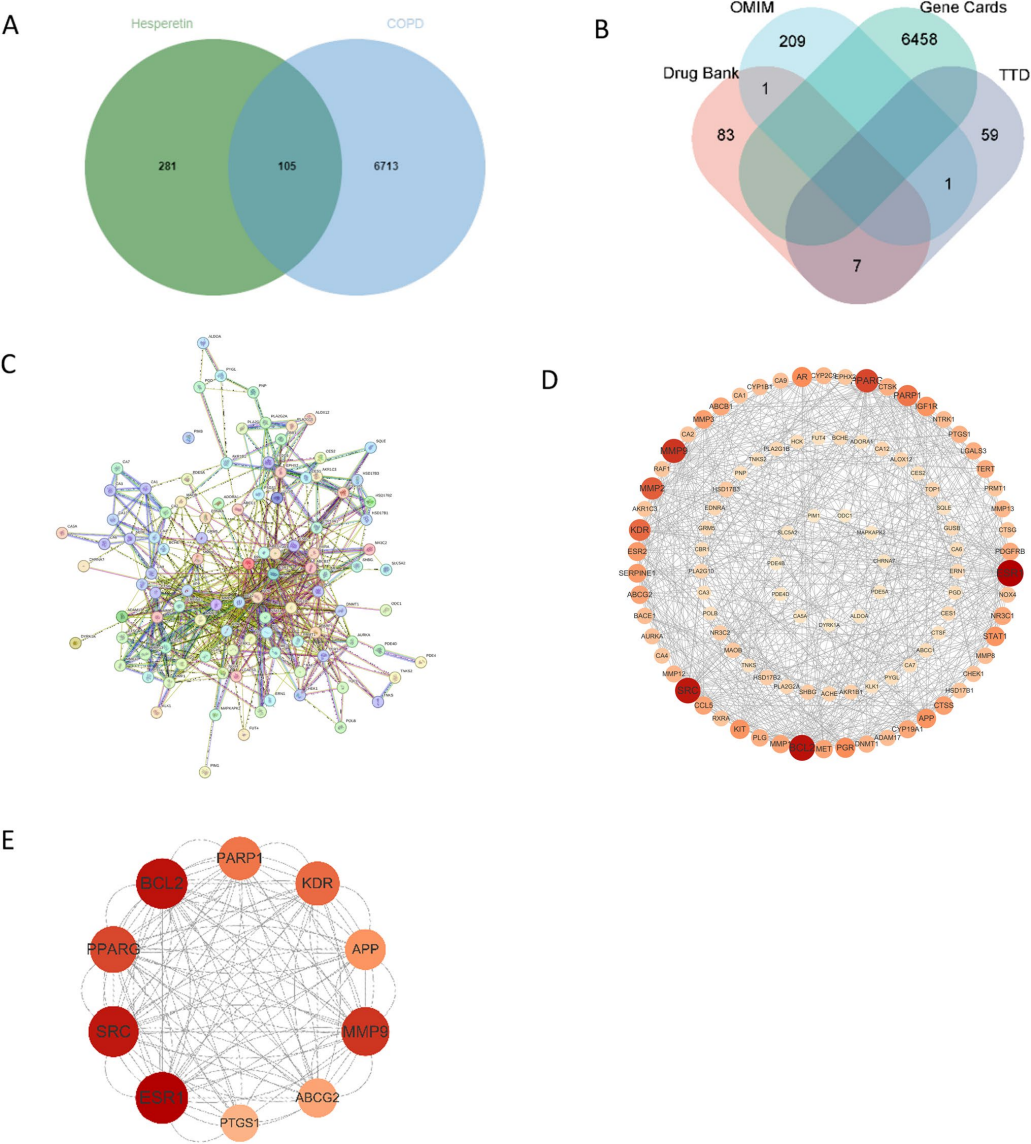
(**A**) Intersection targets of HE and COPD. (**B**) Venn diagram. (**C**) PPI network diagram. (**D**) Construct the interaction of COPD and HE targets using Cytoscape. (**E**) Top ten targets of PPI network analysis.

We used the JVENN platform (https://jvenn.toulouse.inrae.fr/app/example.html) to construct a Venn diagram and derive the intersecting targets (Fig. 3A). It was demonstrated that 105 gene targets potentially contribute to the process through which HE impacts COPD. The Venn diagram illustrates the quantity of targets within each database along with the intersecting genes (Fig. 3B). The PPI network for the intersecting targets was analyzed using the STRING database, revealing a network of 102 nodes and 612 edges (Fig. 3C). The resulting data was then imported into Cytoscape3.91 to build the intersecting target network diagram of COPD and HE (Fig. 3D). Darker color shades for the nodes, as well as larger color differences between nodes and edges, signal stronger connections between proteins. A screening revealed the top 10 core targets to be: Estrogen Receptor 1 (ESR1), Sarcoma Gene (SRC), Peroxisome Proliferator Activated Receptor Gamma Gene (PPARG), B-cell Lymphoma-2 (BCL2), Poly (ADP-ribose) Polymerase 1 (PARP1), Kinase Insert Domain Receptor (KDR), Amyloid Precursor Protein (APP), Matrix Metalloproteinase 9 (MMP9), and ATP Binding Cassette Subfamily G Member 2 (ABCG2) (Fig. 3E).

### GO and KEGG functional enrichment analysis

**Fig. 4:**
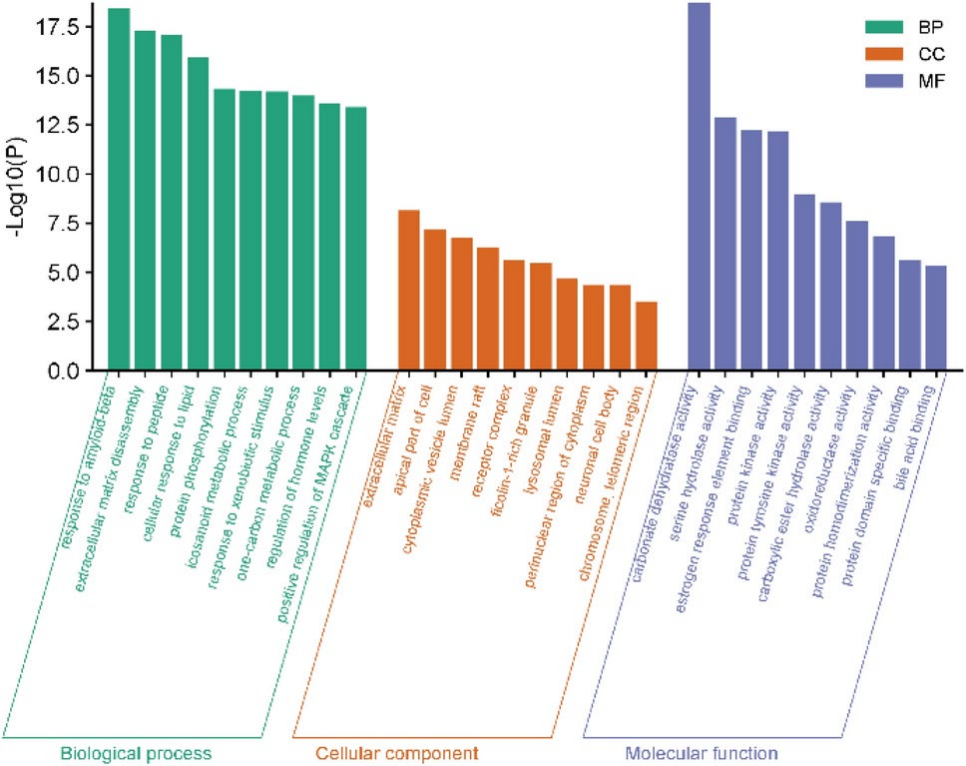
GO pathway enrichment analysis diagram.

To elucidate the functional and pharmacological mechanisms of HE, the intersecting targets were subjected to enrichment analysis using the Metascape database. According to the criterion of *p* < 0.05, 56 biological functions were selected, which included 20 biological processes (BP), Molecular Functions (MF), and 16 Cellular Components (CC). The top 10 significantly enriched BP, MF, and CC categories were arranged in ascending order of p-value, and these biological functions were then visualized (Refer to Fig. 4). Analysis revealed that BP predominantly involved functions related to protein phosphorylation, positive regulation of MAPK signaling cascades, responses to lipids, and responses to amyloid-beta protein. On the other hand, CC primarily involved elements such as isomeric neuronal cell bodies, receptor complexes, extracellular matrix, and additional neuronal cell bodies. MF was mainly concerned with the activities of carbonate dehydratase, serine hydrolase, protein kinase, and protein tyrosine kinase. This indicated that the role of HE in intervening with COPD resulted from multiple effects.

**Fig. 5:**
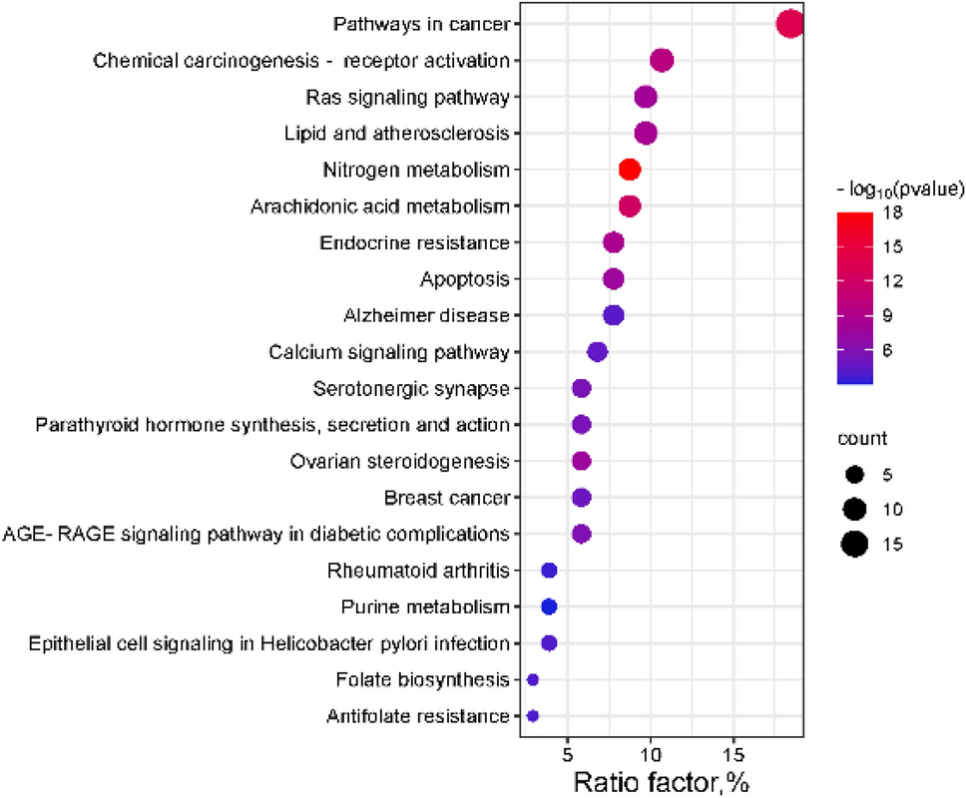
KEGG pathway enrichment analysis diagram.

KEGG enrichment analysis was conducted through the Metascape database, resulting in the identification of 20 related signaling pathways (*p* < 0.01). A bubble map of these signaling pathways was constructed, arranging them in ascending order of p-values (see Fig. 5). The size of each bubble corresponds to the number of enriched pathways, and its color represents the magnitude of the p-value. The KEGG pathway encompasses the Ras, Ca^2+^, and MAPK signaling pathways, which are involved in processes such as nitrogen metabolism, arachidonic acid metabolism, endocrine resistance, and lipid and arteriosclerosis. Upon comparison with the pathway target information in the KEGG database, it was revealed that Ras maintains a linear relationship with NF-κB and MAPKs. This insight guided the subsequent selection of the pathway for experimental verification.

### Molecular docking

**Fig. 6:**
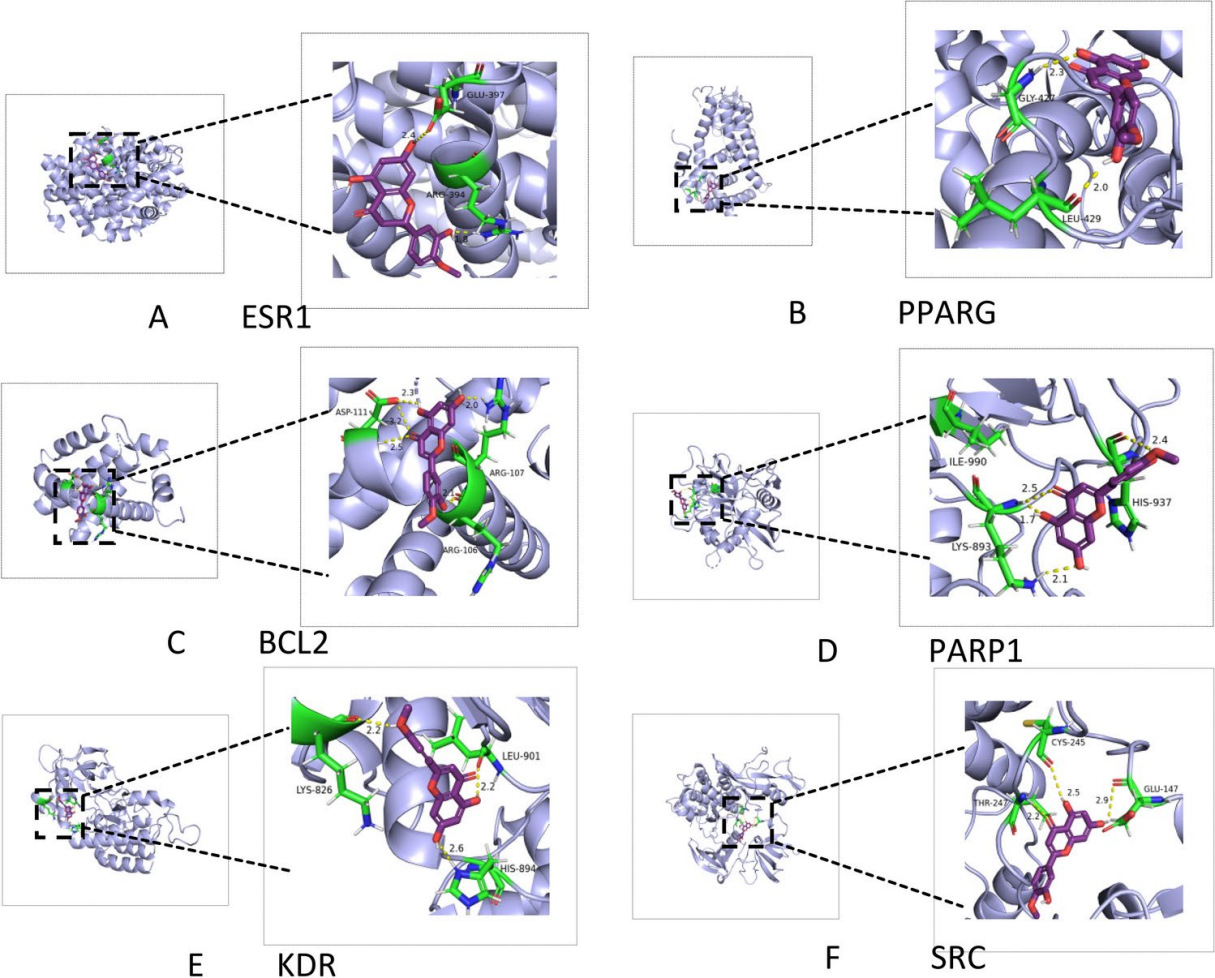
(**A**–**F**) Molecular docking results for ESR1, PPARG, BCL2, PARP1, KDR, and SRC.

The molecular docking results indicated that HE showed strong interactions with six target proteins: ESR1, PPARG, BCL2, PARP1, KDR, and SRC (Fig. 6). The binding affinities of these proteins were less than − 7 kcal/mol, suggesting that they could effectively bind to HE. Table 1 shows the binding energy and hydrogen bond sites of HE with core targets.

**Table 1:** The binding energy and hydrogen bond sites of HE with core targets.

Gene	Binding energy (kcal/mol)	Hydrogen bond sites
ESR1	− 7.4	GLU-397, ARC-394
PPARG	− 8.1	GlY-427, LEU-429
BCL2	− 8.2	ASP-111, ARG-107, ARG-106
PARP1	− 8.8	ILE-990, HIS-937, LYS-893
KDR	− 9.7	LYS-826, LEU-901, HIS-894
SRC	− 9.2	CYS-245, THR-247, GLU-147

The binding energy of the ESR1 protein with HE was − 7.4 kcal/mol, and ESR1 interacted with HE through amino acid residues GLU-397 and ARC-394. The binding energy of the SRC protein with HE was − 9.2 kcal/mol and this interaction was dependent on GYS-245, THR-247, and GLU-147. Residues such as GLY-427 and LEU-429 are necessary for PPARG to interact with HE. BCL2 protein binds to HE utilizing residues such as ASP-111, ARG-106, and ARG-107. The binding energy between PARP1 and HE was − 8.8 kcal/mol, which depended on the interaction of ILE-990, HIS-937, and LYS-893 residues. LEU-901/HIS-894 and LYS-826 residues of KDR bind to HE via hydrogen bonds.

### Animal experiments

#### Effects of HE on pulmonary function

**Fig. 7:**
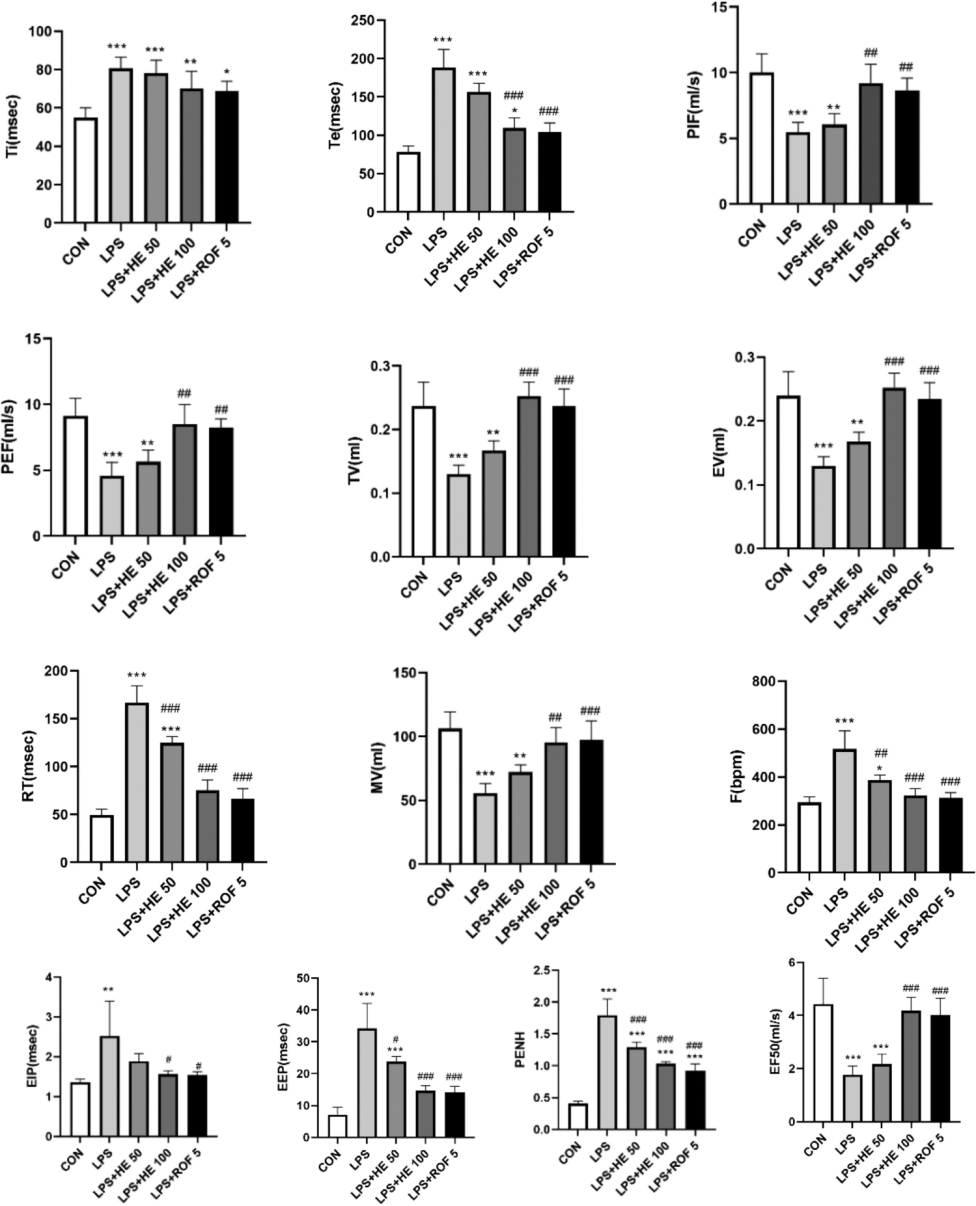
The effects of HE on lung function indices, data presented as Mean ± SD, N = 8, *p < 0.05, **p < 0.01, ***p < 0.001 for comparison with the CON group. ^#^*p* < 0.05, ^##^*p* < 0.01, ^###^*p* < 0.001 indicates comparison with LPS Group.

Compared to the CON group, the lung function indices of the LPS group significantly decreased (Fig. 7). When comparing CON, there were no significant differences in PIF, TV, PEF, EV, MV, RT, F, EIP, EEP, and EF50 between the high-dose HE group and the ROF group. This is indicative that HE is effective in improving lung function injuries caused by LPS.

#### Effects of HE on inflammatory levels

**Fig. 8:**
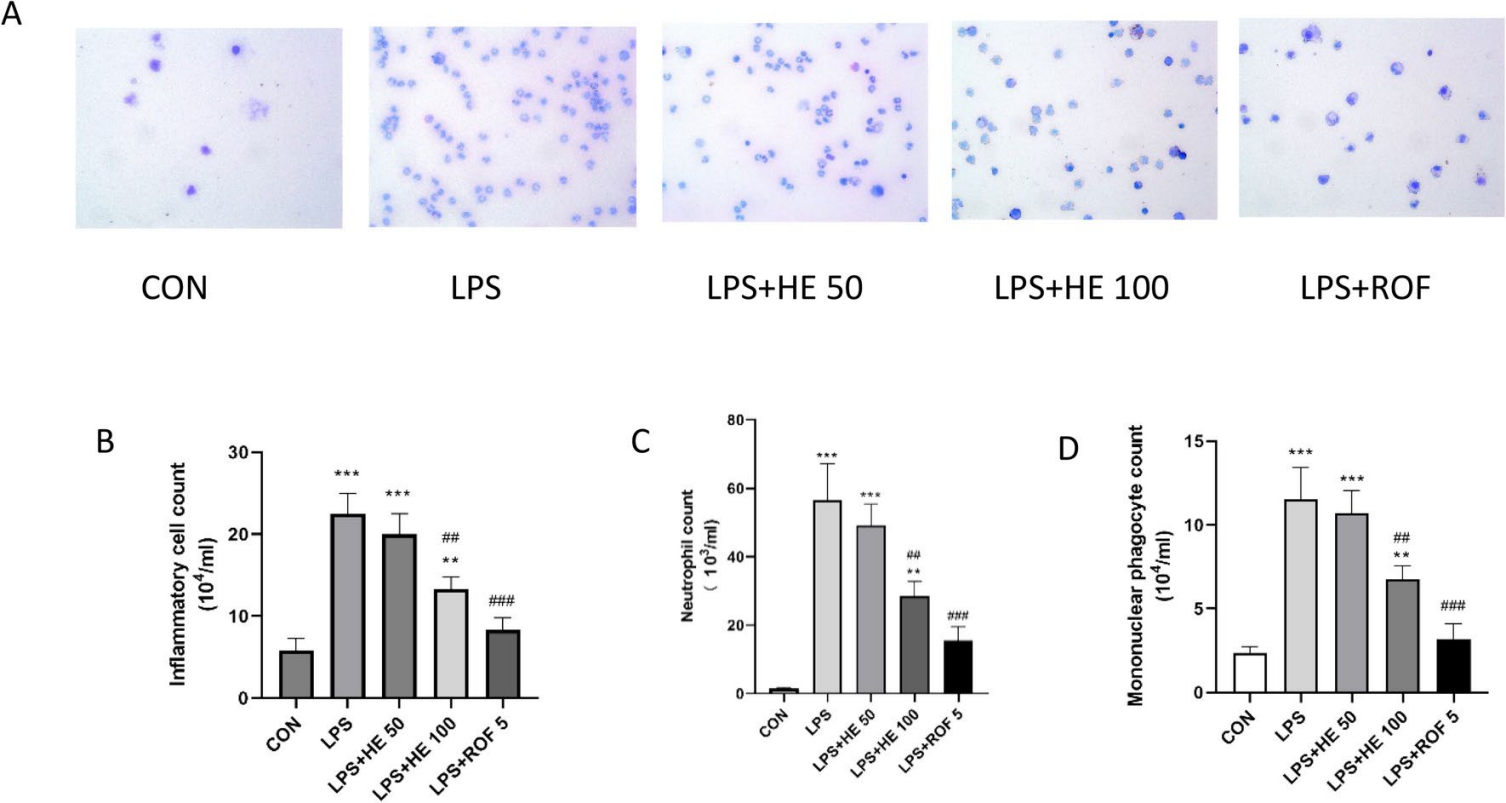
Total number of inflammatory cells, neutrophil cells, and mononuclear giant cells in BALF, data presented as Mean ± SD, N = 8. **p < 0.01, ***p < 0.001 indicates a comparison with the CON group. ^##^*p* < 0.01, ^###^*p* < 0.001 indicates comparison with LPS Group.

**Fig. 9:**
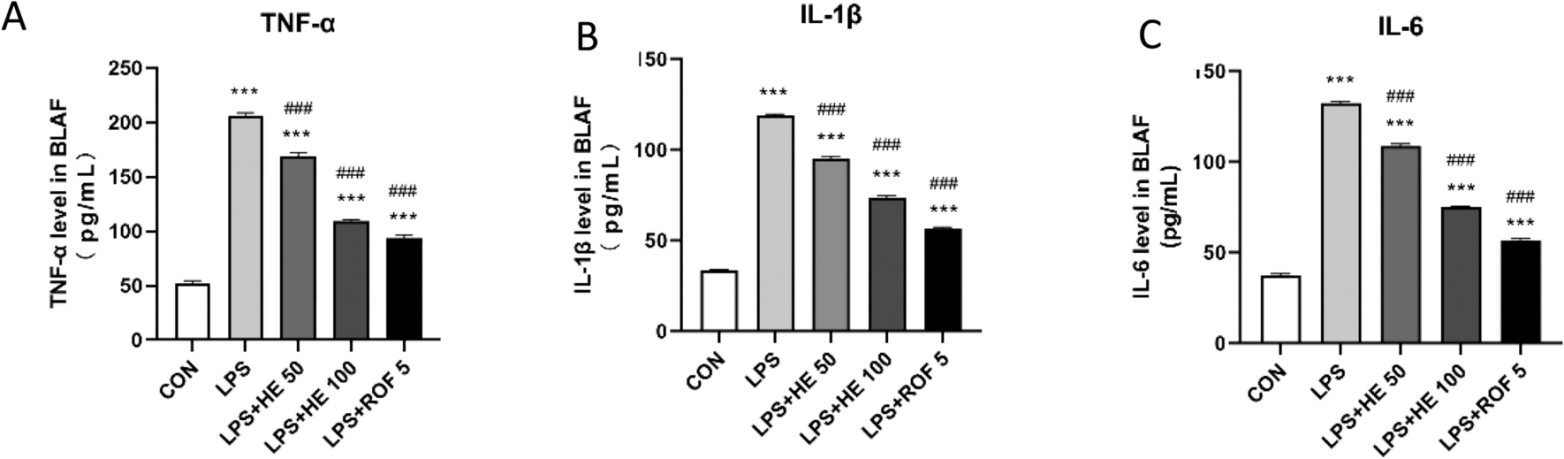
Levels of TNF-α, IL-1β and IL-6 in BALF. Values are expressed as Mean ± SD, N = 8. ^***^p < 0.001 indicates comparison with the CON group; ^###^*p* < 0.001 indicates comparison with the LPS Group.

Compared to the CON group, the LPS group revealed a significant increase in both the number of inflammatory cells and the concentrations of inflammatory mediators TNF-α, IL-6, and IFN-γ. On the other hand, both the LPS + HE 100 and LPS + ROF 5 groups displayed noticeably lower counts of inflammatory cells and decreased levels of inflammatory factors. HE enhanced the LPS-induced inflammatory response, as demonstrated in Figs. 8 and 9.

#### Effects of HE on NE and ROS levels

**Fig. 10:**
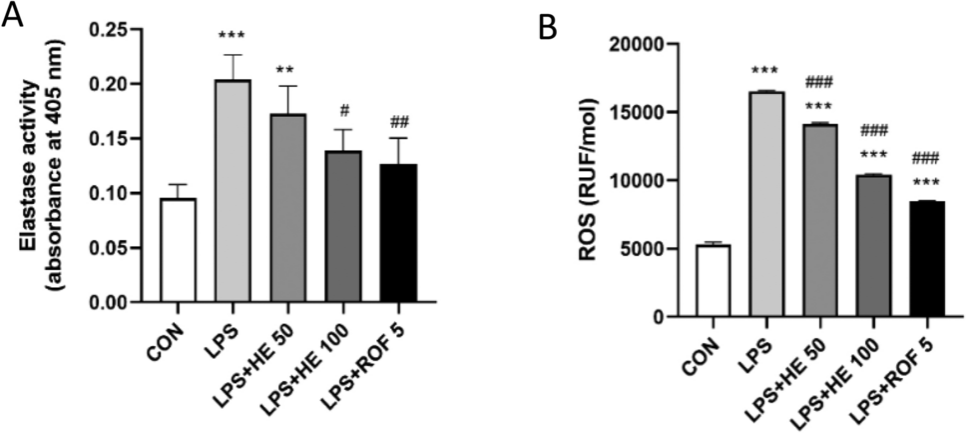
(**A**) NE activity in BALF; (**B**) Levels of ROS in lung tissues. Values are presented as Mean ± SD, N = 8. **p < 0.01, ***p < 0.001 indicates comparison with the CON group; ^#^*p* < 0.05, ^###^*p* < 0.001 indicates comparison with the LPS Group.

Compared with the CON group, NE activity significantly increased in the LPS group (Fig. 10A). This suggests that LPS induction could influence NE activity. On the other hand, upon HE treatment, NE activity notably decreased in the LPS + HE 100 and LPS + ROF 5 groups (*p* < 0.05). This data suggests that high-dose HE and the positive control drug, ROF, could improve NE activity.

Measurements of ROS levels in lung tissue revealed a significant increase in mice following LPS induction (Fig. 10B). However, both HE and ROF significantly contributed to redox recovery. This was evidenced by the significantly lower ROS contents in the LPS + HE 50, LPS + HE 100, and ROF groups compared to the LPS group (*p* < 0.001).

#### Effects of HE on pathological tissues

**Fig. 11:**
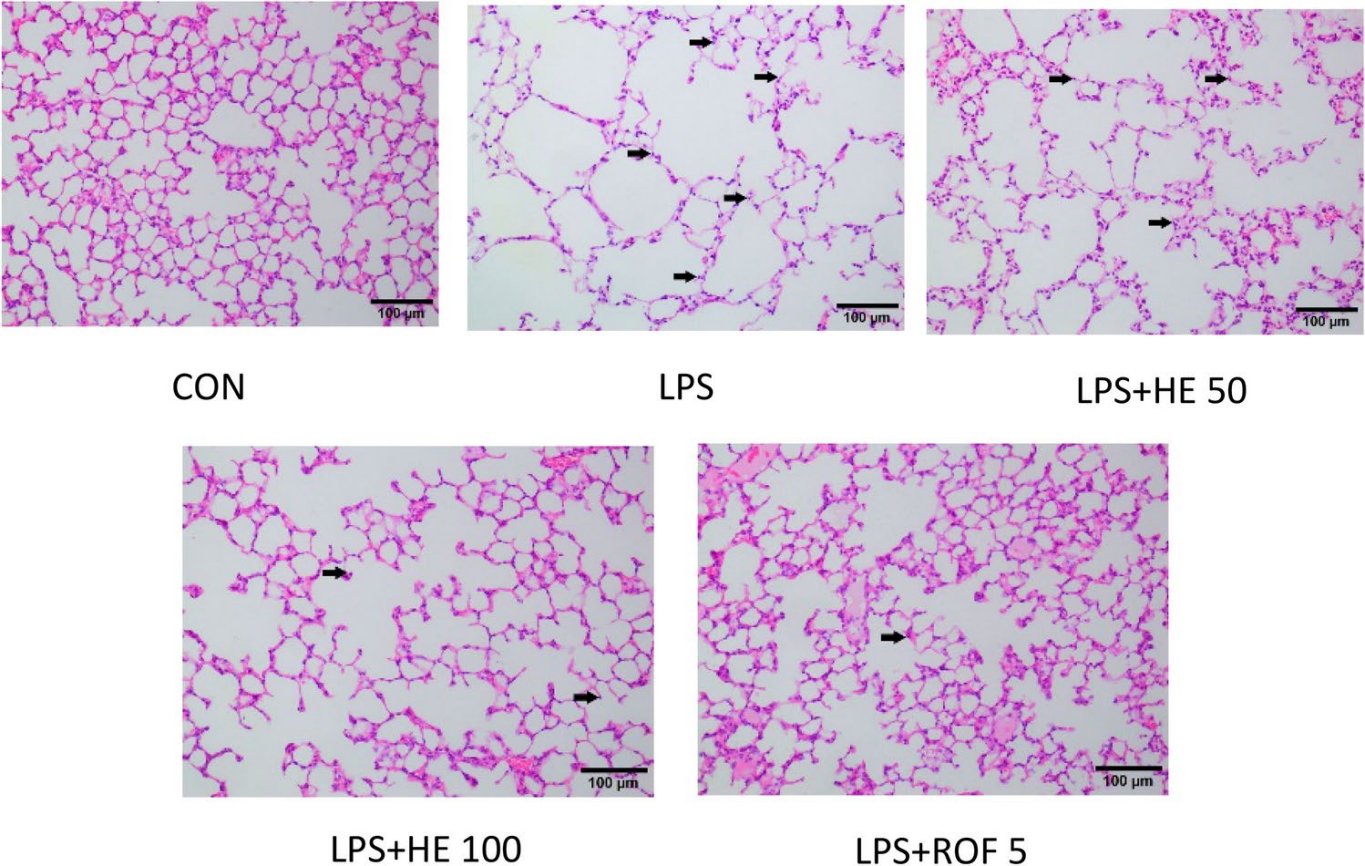
The results of H&E staining of lung tissue in each group (100 ×).

In the CON group, the epithelial cells were well-arranged, and no inflammatory cells were found in the lung. The size of the smooth muscle did not increase, the structure of the alveoli remained intact, and their size was uniform. In the LPS Group, we noted severe inflammatory cell infiltration, proliferation of smooth muscle cells, enlargement of the alveolar space, thinning of alveolar walls, and the presence of alveolar lesions. Compared to the LPS Group, the LPS + HE 100 Group, and the LPS + ROF 5 Group showed less pathological damage and fewer infiltrations of inflammatory cells (see the arrow in Fig. 11).

#### Effects of HE on the MAPKs/NF-κB signaling pathway

**Fig. 12:**
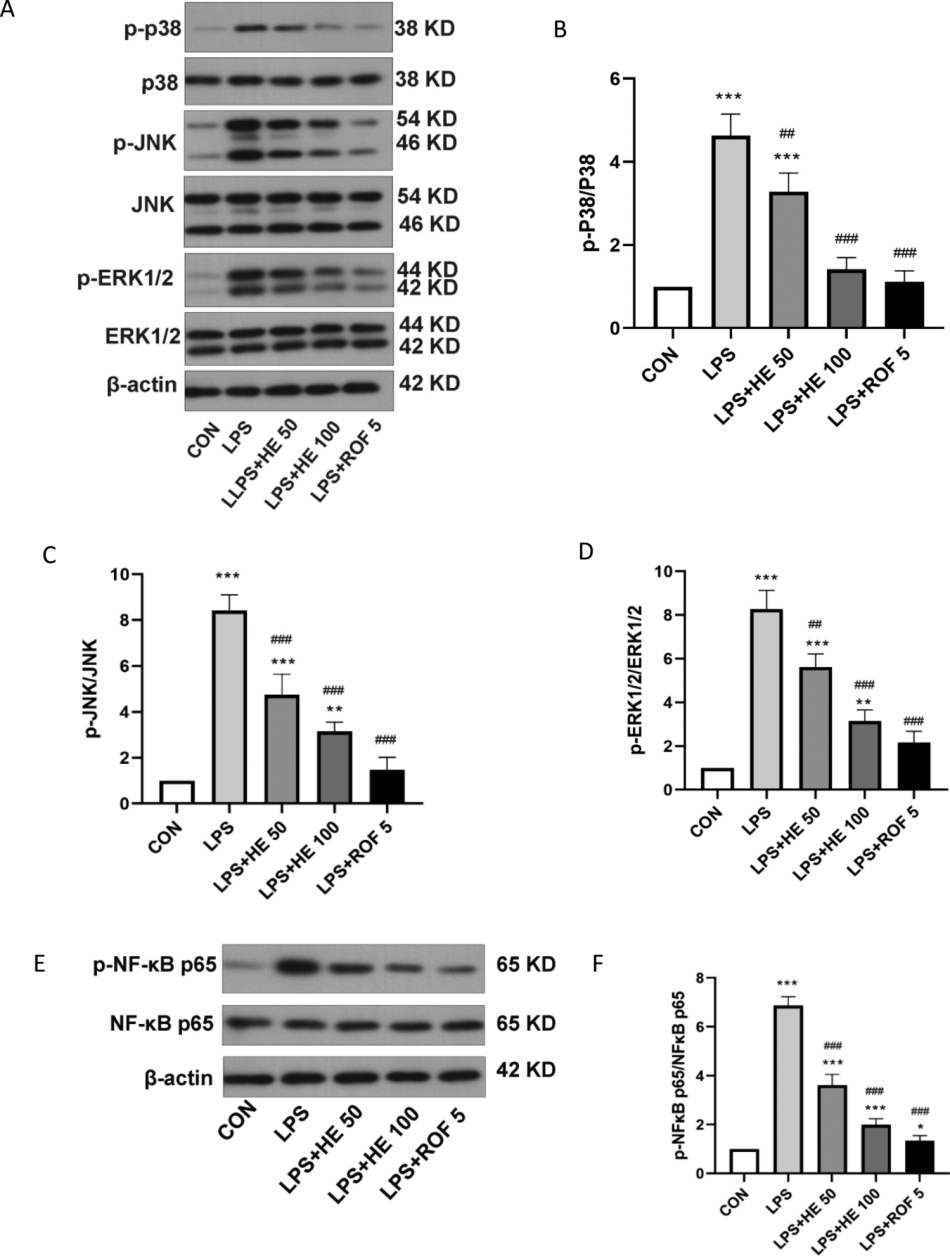
Effects of HE on MAPKs and NF-κB signaling pathways. (**A**–**D**) WB analysis of p-p38, p-JNK and P-ERK1/2 pathways and (**E**–**F**) WB analysis of p-NF-κB P65 pathway. Data presented as mean ± SD, N = 4. **p < 0.01, ***p < 0.001 indicates comparison with the CON group; ^#^*p* < 0.05, ^###^*p* < 0.001 indicates comparison with the LPS Group.

To investigate the roles of the MAPKs and NF-κB pathways in LPS-induced COPD in mice, we used WB analysis to evaluate the expression of proteins related to these signaling pathways. As depicted in Fig. 12B–E, LPS significantly increased the phosphorylation levels of p38, ERK1/2, JNK, and NF-κB p65 compared to the CON group. This was indicated by the ratios of p-p38/p38, p-ERK1/2/ERK1/2, p-JNK/JNK, and p-NF-κB p65/NF-κB p65. However, these augmented effects were mitigated with HE treatment, with high-dose HE exhibiting a similar effect to that of the ROF group (Fig. 12A–F). These results suggest that HE may inhibit the progression of COPD by suppressing the activation of both the MAPKs and NF-κB signaling pathways.

## Discussion

The purpose of this study is to examine the potential targets and pharmacological mechanisms that contribute to the therapeutic effects of HE in addressing COPD. In traditional Chinese medicine (TCM), HE is believed to treat cough, phlegm, and upper abdominal distension^44^. Furthermore, numerous studies underscore that HE, a naturally occurring compound, possesses anti-inflammatory and antioxidant properties. It is capable of reducing inflammation and improving lung function in COPD patients through the regulation of inflammatory signaling pathways and the inhibition of oxidative stress responses^45–47^.

We identified 105 intersection targets shared by COPD and HE, deemed as potential gene targets for COPD treatment with HE. The PPI network map (Fig. 3E) underscores the top ten targets; ESR1 is not only connected to estrogen receptors but it also shows associations with lung function and lung inflammation^48,49^. The SRC family, comprising 11 members, is a receptor tyrosine kinase family playing a vital role in intracellular signal transduction and the control of inflammatory responses^50^. It can orchestrate cell proliferation, differentiation, invasion, and metastasis via MAPK, PI3K, STAT3, and other signaling pathways^51^. BCL2 can modulate the intracellular calcium concentration, preserve the regular function of cells, and decrease the stress response related to apoptosis^52^. To validate if HE can serve as a pivotal compound for the treatment of COPD, we conducted molecular docking on the core targets (Fig. 6). The results showed that HE has a strong binding activity to ESR1, PPARG, BCL2, and other key targets. Consequently, we hypothesize that HE can be utilized as an active ingredient of TCM for COPD treatment.

Go enrichment analysis demonstrated that HE exerts anti-inflammatory, oxidative stress-inhibitory, and immunomodulatory effects by regulating the positive regulation of MAPK cascades, protein phosphorylation, signaling, and cell proliferation. The KEGG enrichment analysis indicated that the core targets’ primary involvement in the PPI network is in signaling pathways, such as those related to Ca^2^⁺ and Ras. Notably, the Ras signaling pathway exhibits a clear linear relationship, both upstream and downstream, with NF-κB and MAPKs. Additionally, numerous studies have shown that the NF-κB signaling pathway plays a significant role in lung inflammation and oxidative stress^47,53–55^. The activation of the MAPK cascade is central to a variety of signaling pathways^56^, and oxidative stress and inflammatory responses during COPD can be suppressed by regulating the SRC/MAPK pathway^57^. Therefore, the NF-κB and MAPK signaling pathways were identified as key signaling pathways for conducting animal studies.

A pulmonary function test is a key tool for evaluating COPD^58^. Moderate-to-high flavonoid intakes were associated with a lower risk of COPD and better lung function, particularly among ever smokers. Promoting intakes of healthy flavonoid-rich foods, namely, tea, apples, and berries, may improve respiratory health and lower COPD risk, particularly in individuals with a smoking history^59^. In our study, we measured 13 indices linked to pulmonary function and found that the high-dose HE group was effective in ameliorating lung function injury sparked by LPS (Fig. 7). A further examination revealed a spike in the count of inflammatory cells, including neutrophil and macrophage cells, in the BALF of LPS-treated mice. However, the recruitment of these inflammatory cells decreased following HE treatment, suggesting that HE could counteract the inflammatory response induced by LPS.

The cytokine TNF-α is closely related to the promotion of airway remodeling and exacerbation of airflow limitation^60^. IL-1β plays a crucial role in the recruitment and activation of neutrophils^61^. The multifunctional cytokine IL-6 not only stimulates inflammation but also plays a vital role in immune regulation, amplifying chronic inflammation and contributing to imbalances in the immune system^62^. Our study found that in the lung tissue of LPS-treated mice, the levels of TNF-α, IL-1β, and IL-6 were increased compared to the control group (Fig. 9). However, in the LPS + HE 100 group treated with HE, the levels of TNF-α, IL-6, and IL-1β in the lung tissue were significantly higher than those in the control group. Despite this increase, we observed that the secretion of inflammatory cytokines was inhibited. This suggests that HE has a potential therapeutic effect in alleviating the LPS-induced inflammatory response.

In COPD, neutrophil inflammation can lead to further lung tissue damage. This process involves the release of several proteolytic enzymes, including NE^63^. The results revealed that the activities of NE and ROS were significantly enhanced following LPS induction in the mouse model, while NE activity decreased after HE treatment. These results indicate that HE contributed to REDOX recovery and reduced the production of LPS-stimulated intracellular ROS. This is in line with the findings of Ana Beatriz Farias de Souza et al.^64^. To further evaluate the effect of HE on lung injury in mice, we observed changes in histopathology. H&E staining revealed that HE significantly improved the pathological lesions and reduced inflammatory cell infiltration.

Building on the results of our network pharmacological analysis, we further validated the roles of the MAPK and NF-κB signaling pathways through mouse experiments. The MAPK pathways include P-38, ERK1/2, and members of the JNK family. We have observed that the expression of p-p38/P-38, P-ERK1/2/ERK1/2, p-JNK/JNK, and p-NF-κB p65/ NF-κB p65 were notably inhibited following HE treatment. The JNKs belong to the family of mitogen-activated protein kinases, which regulate many physiological processes. Numerous studies have indicated that the JNK signaling pathway plays a role in COPD pathogenesis through the regulation of inflammatory responses^65,66^. Additionally, exposure to cigarette smoke markedly increases JNK phosphorylation levels^67^ and can simultaneously upregulate p38 activation and expression, which subsequently stimulates pro-inflammatory cytokine and chemokine production^68^. The NF-κB signaling pathway can also stimulate pro-inflammatory cytokine production. Previous studies have shown that improvements in COPD are closely linked to the inhibition of NF-κB^69^. These findings align with our experimental results. Hence, it is suggested that the anti-inflammatory effect of HE may be realized by inhibiting the activation of the NF-κB and MAPK signaling pathways.

Despite certain limitations in this study, such as the huge number of small molecular compounds in TCM ingredients and the considerable workload involved, only a limited number of active compounds could be identified through data screening based on established criteria. Additionally, the accuracy and comprehensiveness of the database need improvement. Nevertheless, the subsequent animal experiments conducted in this study provide a valuable resource for elucidating the mechanisms of action of HE in treating COPD.

## In conclusion

In this study, we utilized network pharmacology, molecular docking, and experimental validation to investigate the potential target genes and mechanism through which HE produces its effects in treating COPD. The results showed that HE could enhance lung functionality and pathological morphology in COPD cases, and lower inflammation levels. This improvement may be connected to the inhibition of MAPKs and NF-κB signaling pathways.

## Supplementary Information

Supplementary Information 1.

Supplementary Information 2.

Supplementary Information 3.

## Data Availability

The datasets presented in the study are included in the article/Supplementary Material, further inquiries can be directed to the corresponding authors.

## Abbreviations

COPD: Chronic obstructive pulmonary disease
HE: Hesperetin
ROF: Roflumilast
LPS: Lipopolysaccharide
BALF: Bronchoalveolar lavage fluid
ROS: Reactive oxygen species
NE: Neutrophil elastase
GO: Gene ontology
KEGG: Kyoto encyclopedia of genes and genomes
TNF-α: Tumor necrosis factor-alpha
IL-6: Interleukin-6
IL-1β: Interleukin-1 beta
MAPKs: Mitogen-activated protein kinases
NF-κB: Nuclear factor-κB
TI: Inspiratory time
PIF: Peak inspiratory flow
TV: Tidal Volume
EEP: End expiratory pause
TE: Expiratory time
PEF: Peak expiratory flow
EV: Expiratory volume
EIP: End-inspiratory pause
F: Frequency
RT: Relaxation time
PENH: Enhanced pause
MV: Minute ventilation volume
EF50: Expiratory flow at 50%
H&E: Hematoxylin–eosin
ELISA: Enzyme-linked immunosorbent assay
WB: Western blot
ANOVA: One-way analysis of variance
TCM: Traditional Chinese Medicine
