# Supplementary material for: Integrated network pharmacology, molecular docking, and animal experiments to reveal the potential mechanism of hesperetin on COPD
Source: Sci Rep. 2025 Apr 1;15:11024. doi: 10.1038/s41598-025-95810-4 (PMC11958725; doi:10.1038/s41598-025-95810-4)
Supplement: Supplementary file 3 — Supplementary Information 3. [file 41598_2025_95810_MOESM3_ESM.pdf]

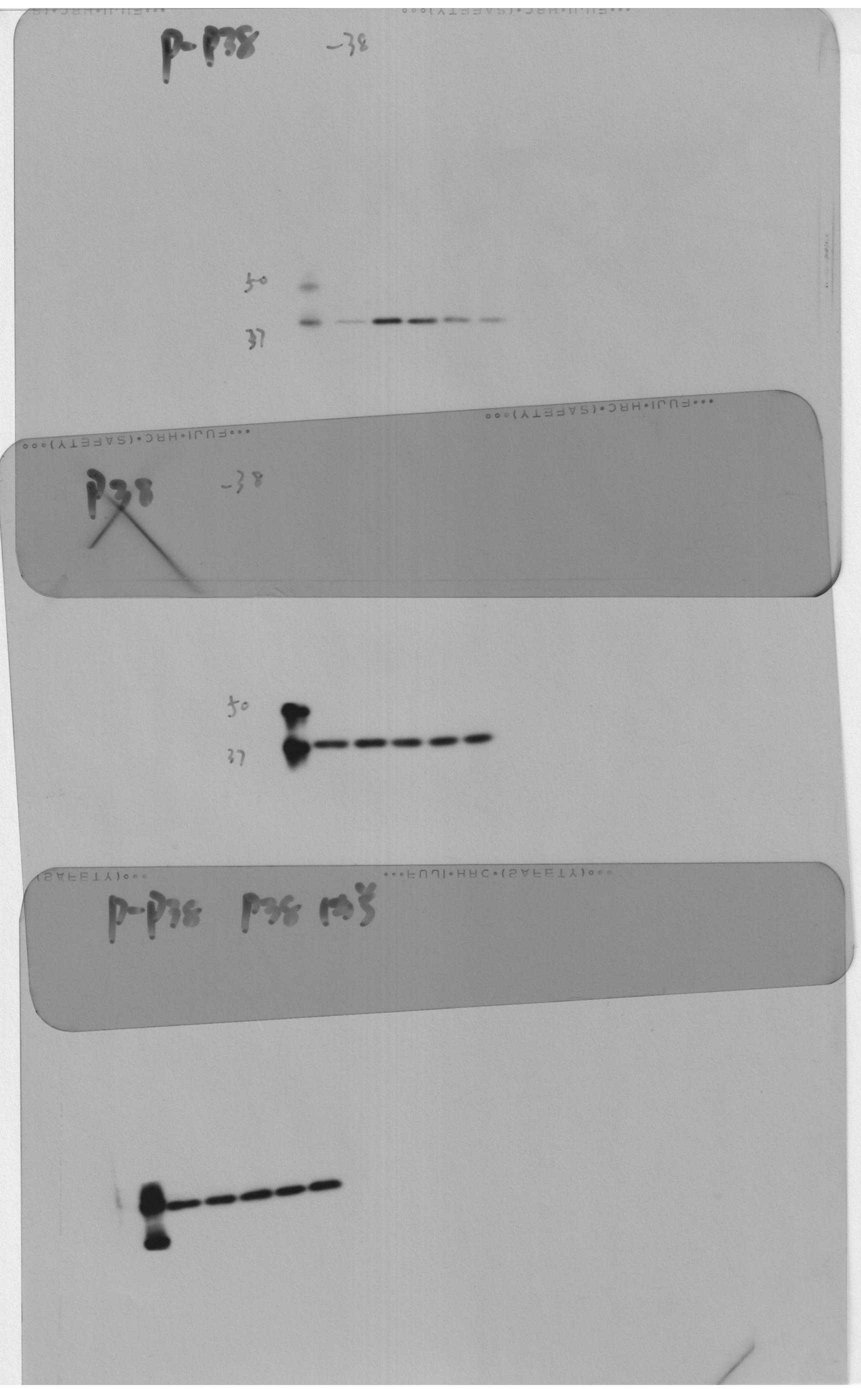
p-p38
50
37
p38
50
37
p-p38 p38

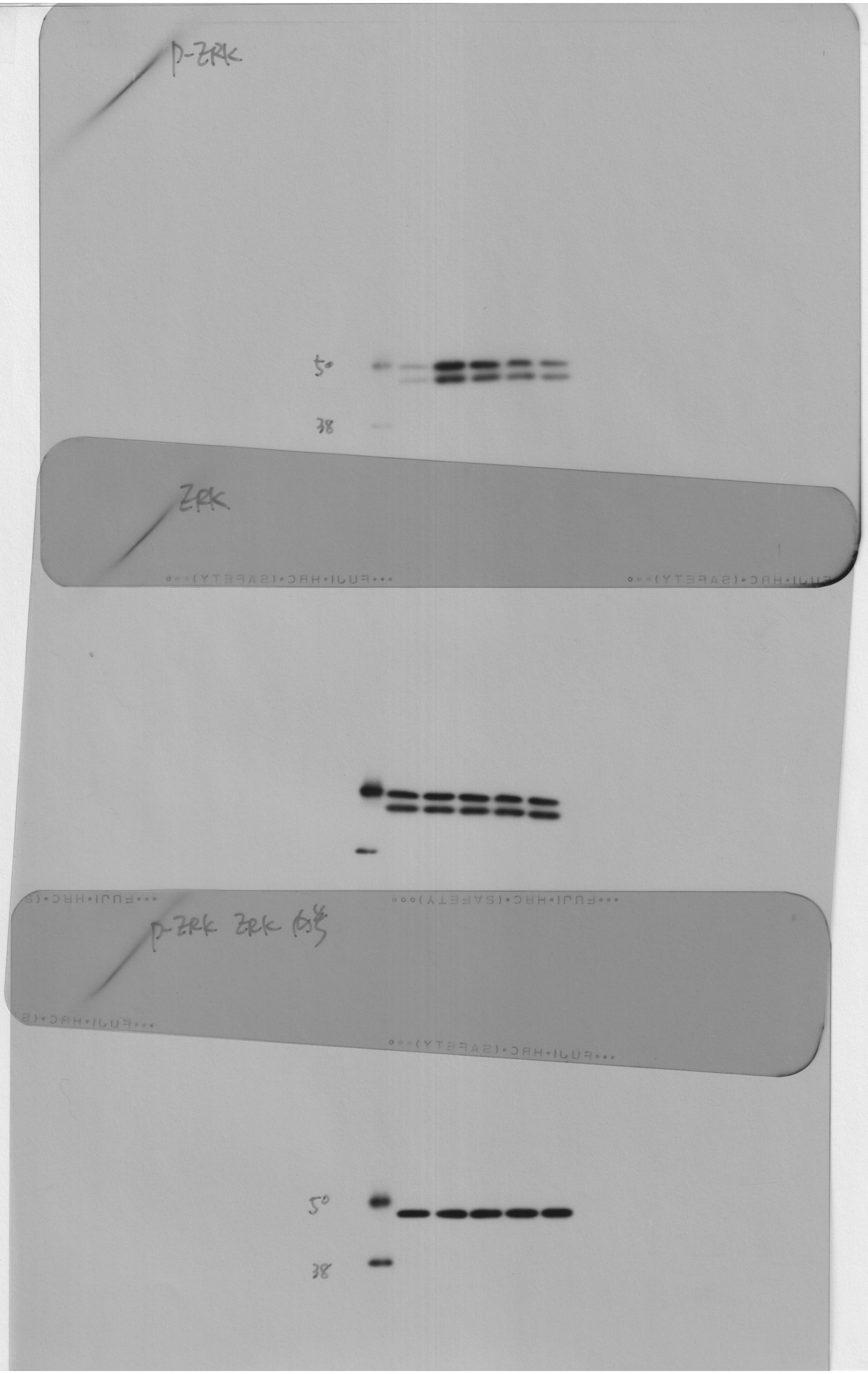
p-ERK
50
38
ERK
p-ERK ERK
50
38

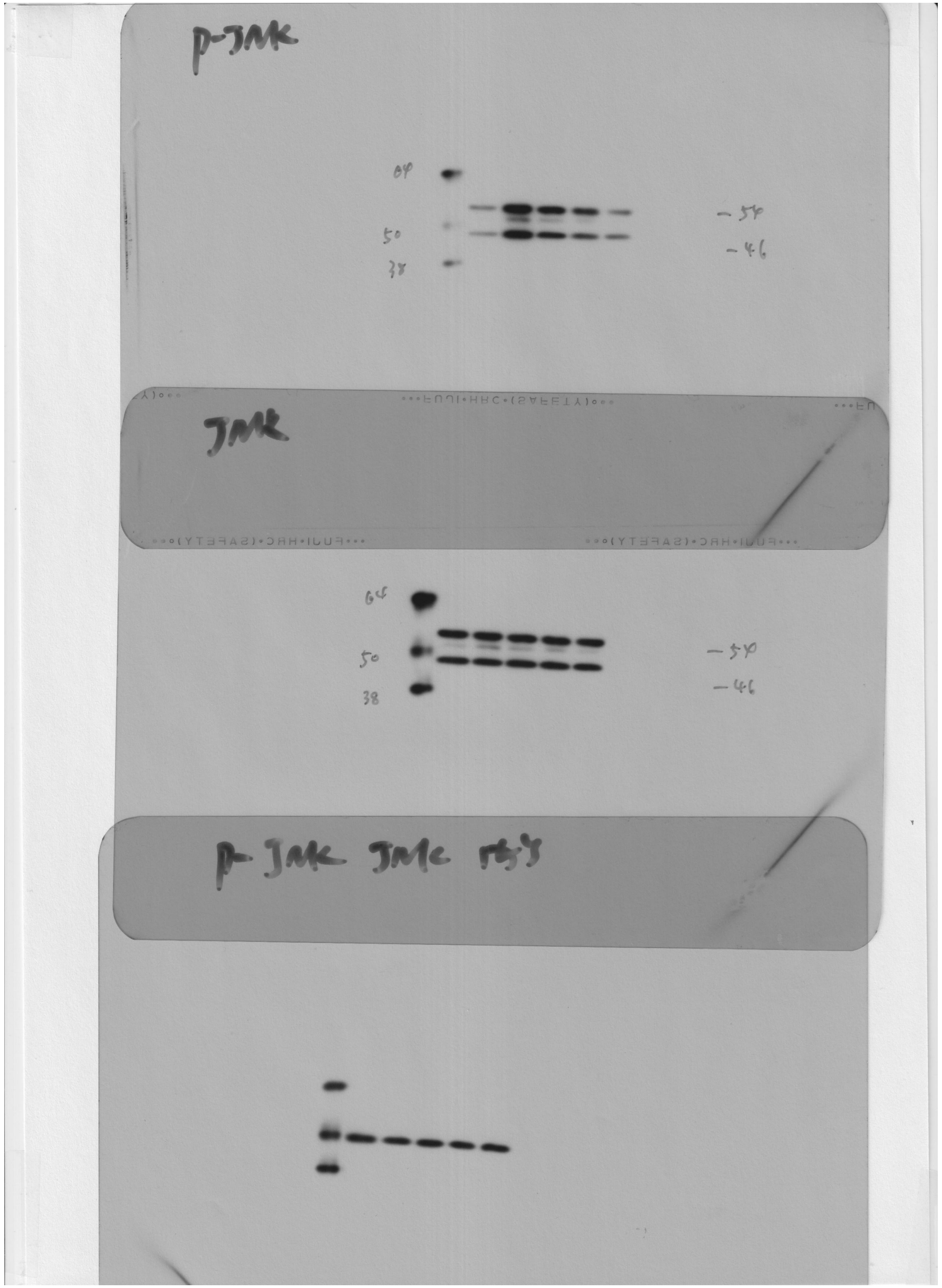
p-JAK
64
50
38
— 54
— 46
JAK
64
50
38
— 54
— 46
p-JAK JAK

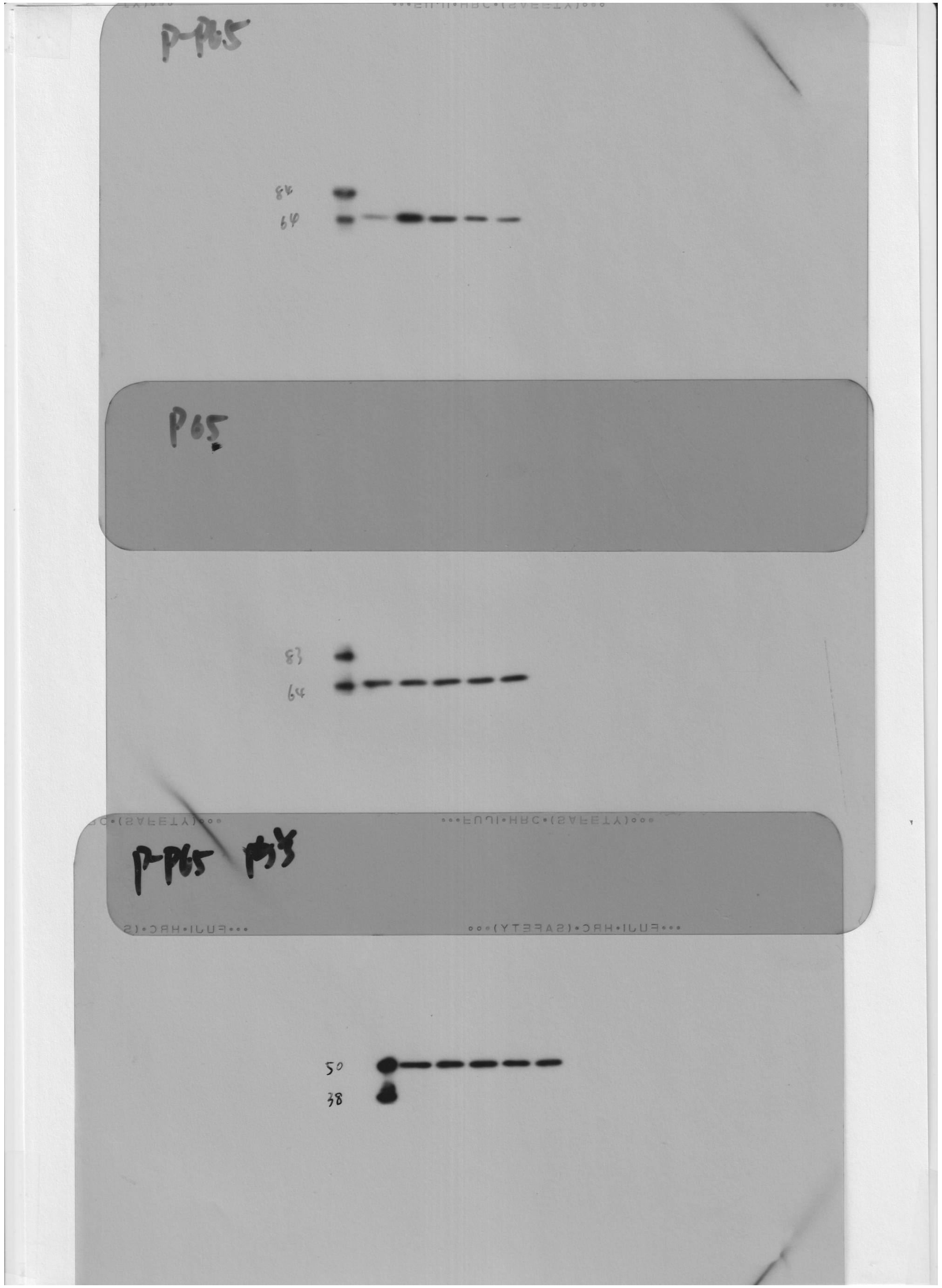
p-P65
P65
p-P65
50
38
